# Adult education needs inventory: Construction and application

**DOI:** 10.3389/fpsyg.2022.1035283

**Published:** 2022-12-23

**Authors:** Luba Ślósarz, Kamil Błaszczyński, Magdaléna Švecová, Aleksander Kobylarek

**Affiliations:** ^1^Department of Humanities and Social Science, Wroclaw Medical University, Wroclaw, Poland; ^2^Institute of Political Sciences, University of Wrocław, Wroclaw, Poland; ^3^Faculty of Mass Media Communication, University of Ss. Cyril and Methodius in Trnava, Trnava, Slovakia; ^4^Institute of Pedagogy, University of Wroclaw, Wroclaw, Poland

**Keywords:** educational needs, key competences, development of adults, lifelong learning, andragogy

## Abstract

**Background:**

The article presents the psychometric parameters and implementation of the adult education needs inventory (AENI) questionnaire, which is designed to measure the key competences of adult educators. It was constructed on the concept of key competences as proposed by the Council of the European Union. This model of competences is inscribed in the concept of lifelong learning and does not concentrate on the compensatory functions of the competences but merely promotes the insight of educators into their self-development. This article presents the construction process and psychometrical properties of AENI.

**Materials and methods:**

The reliability of the test is confirmed by the inter-correlated results. The accuracy of the questionnaire was confirmed through principal component analysis (PCA). Apart from this, the accuracy of the theory was verified by a correlation between AENI and the Social Skills Profile (PROKOS), which measures the level of such social skills as assertiveness, cooperativeness, sociability, social resourcefulness, and social activeness. To check the accuracy of the theory, hypotheses were formulated which were related to both the correlated overall results (AENI and PROKOS) and the chosen scales.

**Results:**

Six areas of need in development were distinguished: communication skills, multilingual and multicultural skills, digital skills, entrepreneurial skills, openness to science and culture, and social and civic skills. A low result on the scale indicates a need to develop a given skill because the respondent’s skills are weak. Such information is vital for employers and educators who wish to diagnose the areas that need improvement. The measures of reliability and accuracy allow us to state that the questionnaire possesses acceptable psychometrical factors. This study contains propositions for further improvement of the questionnaire and a key to interpreting the research results.

**Conclusion:**

Adult education needs inventory questionnaire is a reliable research tool that can be used to assess the competences of adult educators. Also, it can be considered a voice of discussion regarding the necessity of raising the quality of education and raising the awareness of the education needs of individual adult educators and organizations that benefit from their services.

## 1. Introduction

Lifelong education is typically a comprehensive system, with the entirety and pertinence as the main features, and is composed of multiple elements (Wang et al., 2020). On the one hand, there are still people at risk of exclusion due to deficiencies in primary education. On the other hand, there is a great need to find one’s way in an ever-changing reality characterized by technological advances and globalization (Yamashita et al., 2019). As the modern reality is characterized by the growth of cognitive and informational resources in learning, researchers and theorists of adult education note that there is a global trend away from the concepts of “knowledge” and “skill” to the concept of “competence” (Sin et al., 2020).

An example of the concept of researching educational needs in its broadest sense would be the work of Stufflebeam et al. (2012), which, in turn, is not suitable for analyzing key competence deficiencies among adult students. The problem of classifying key competences for adults who are learning is being resolved by the Council of the European Union. Its latest classification of key competences could become a notional base for testing how far we could make use of a standard, universally accessible tool. A text published in 2018 in the Official Journal of the European Union, with recommendations as to key competences in the process of lifelong learning, presented a classification and definition of key competences which could become the basis for constructing a universal, multidimensional and cohesive concept for research. The proposed model could be used for self-evaluation of adults’ educational needs in the area of key competences, and also extended by the opinions of colleagues and seniors using the technique of 360-degree feedback (or multi-source feedback) in both individual diagnoses and those of the whole workforce (Telling and Serapioni, 2019).

The general concept of educational needs (understood as the presence of disability) has often presented academics with the problem of an appropriate definition due to haziness, ambiguity, and the possibility of a multidimensional approach to the problem (Griffith, 1978). In the literature on the subject, we often come across the term “special educational needs,” which refers to creating specific conditions for atypical students with learning disabilities (Morris, 2001). Other approaches propose the concept of “educational needs assessment,” which generally concentrates on defining educational objectives in specific professions and institutions. However, this term is also used in relation to strictly defined groups of adult students; in other words, it defines the fairly generalized effects of education within a given company environment. In this case, it would be difficult to define some kind of general set of needs, because deficiencies in this area cannot be defined until after a thorough analysis of skill deficiencies among the personnel and the training needs of a particular institution which results from this (Padzik, 2016).

Thus, educational needs can be understood as the necessity of teaching specific skills resulting from an analysis of deficiencies, the replenishment of which will be the objective of a planned education program (Darling-Hammond et al., 2020). However, in the case of adults who already function relatively well in the work environment, understanding their educational needs as being connected with special educational needs would lead to adult education being understood as having a compensatory function (Lodge et al., 2018). In addition, projects are being developed to support adults at risk of exclusion (Pinto et al., 2016). Nevertheless, to a large extent, adult education is not about educational deficiencies; it is about what goals they set for themselves and how they want to use and develop their resources (Panacci, 2015).

In the rapidly developing modern world, a proper function is much more important, which aims to equip people with the skills to understand and change that world and not merely follow it and adapt to it (Joynes et al., 2019).

There are many projects outlining the key competences of adult educators, but there need to be more tools with which to find out whether such competences are present and to what extent. Therefore, the study aimed, in addition to identifying the key competences of adult educators, primarily to develop a way to measure them. The authors wanted the AENI questionnaire developed by them to be widely used by andragogists for the diagnosis and self-diagnosis of key competences helpful in their work.

## 2. Materials and methods

The model proposed by the Council of the European Union regarding lifelong learning was employed as the notional basis for highlighting various content areas. In the 21st century, this model covers the following set of key competences: (1) skills in understanding and composing information; (2) multilingual skills; (3) mathematical skills, and competences in the natural sciences, technology, and engineering; (4) digital skills; (5) personal and social competences concerning learning skills; (6) civic skills; (7) entrepreneurial skills; and 8) competences in cultural awareness and expression (Council of the European Union, 2018).

The statements (*N* = 56) responding to individual areas were developed by considering the following areas of expression: skills, attitudes, and behavior. Next, the list of items was consulted by a panel of experts. These experts were academics with the title of doctor (*N* = 8) working in the field of andragogy, particularly adult education. On a scale of 1–5, the experts assessed the usefulness of individual items in delineating the educational needs of adults. Finally, the assessments of these experts were compared with each other, considering the assessment’s level and integrity. The Cronbach’s alpha test (*α* = 0.97) showed that the internal integrity of the experts’ assessments was very high.

At this stage, none of the items were rejected but edited instead (according to the experts’ guidelines) to increase the statements’ comprehensibility and unambiguity. At this stage, the AENI questionnaire was made up of eight areas, containing 56 items altogether, and had the following structure:

1. Skills in understanding and composing information (8 items),
2. Multilingual skills (8 items),
3. Mathematical skills and competences in the natural sciences, technology and
4. Engineering (8 items).
5. Digital skills (5 items),
6. Personal and social competences concerning learning skills (7 items),
7. Civic skills (6 items),
8. Entrepreneurial skills (9 items),
9. Competences in cultural awareness and expression (5 items).

The 5-point Likert Scale was used to assess individual statements, where 1 = ‘decidedly do not agree’ and 5 = ‘decidedly agree’. The respondents’ personal attributes were established by verifying the following socio-demographic variables: sex (male/female); age; type of organization (state, private or non-governmental organization); and position held within the organization (owner/director, manager, worker, or volunteer).

## 3. Results

### 3.1. Analysis of the scale structure

#### 3.1.1. Description of the research sample

The random sample consisted of 210 participants (28 male and 182 female). The sample size was calculated (Sapra, 2022) based on the GUS survey (2020) that there are 150,000 non-government full-time job positions in Poland. Assuming fraction size on 0.9 and 95% confidence interval, the minimal probe was calculated on 138 observations. Due to the large NGO market in Wrocław, we wanted to increase the probe size when suitable circumstances occur; thus, the final probe has been noted on 210 observations and proved highly representative. Participants were selected based on their engagement in the third sector’s professional, semi-professional, or amateur activities.

#### 3.1.2. Explorational factorial analysis

**Figure 1:**
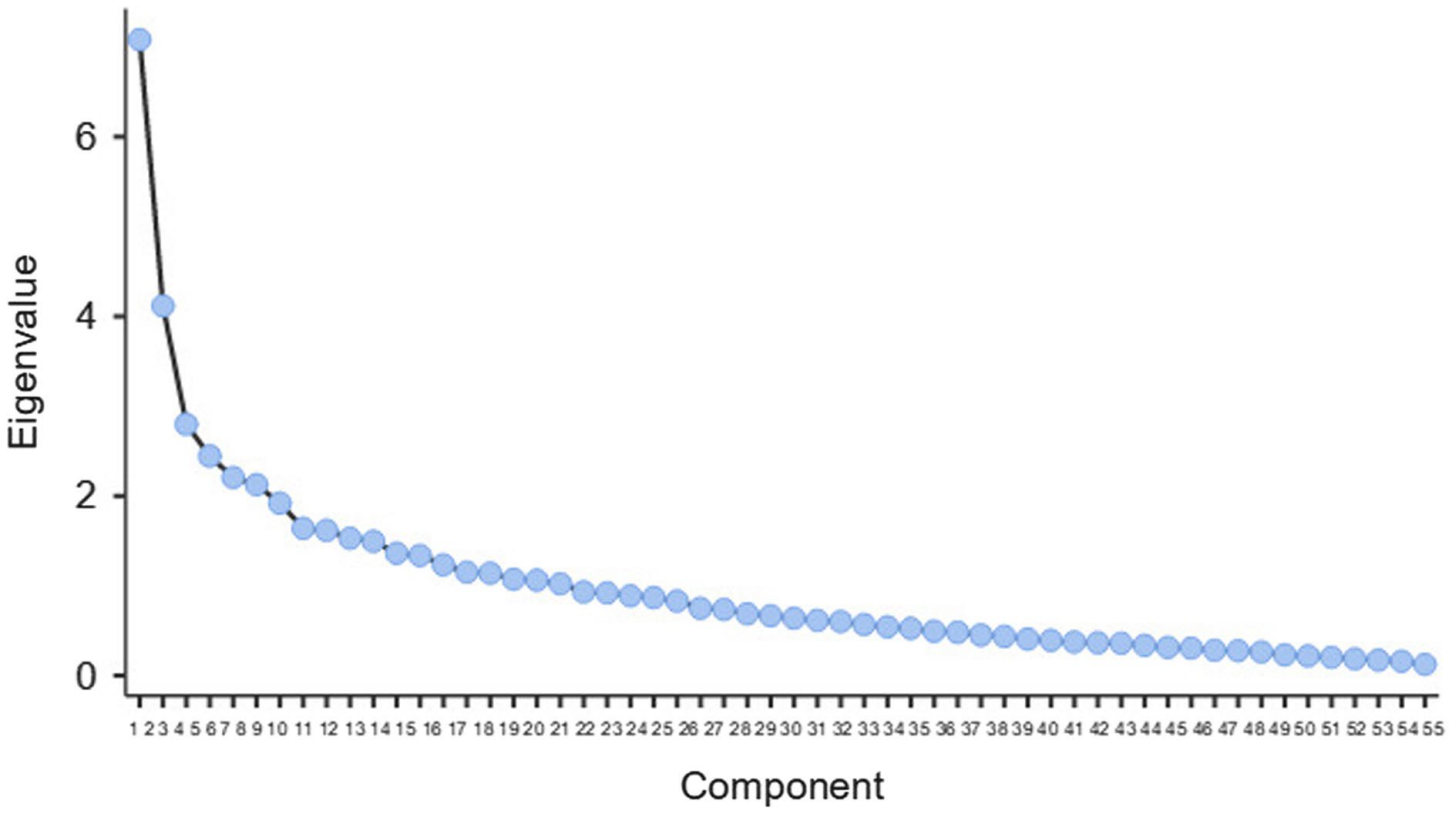
Principal component analysis of the AENI questionnaire. AENI, adult education needs inventory. Component screen plot.

The results of the Kaiser-Meyer-Olkin (KMO) measure of sampling adequacy verified after Varimax rotation (MSA = 0.709) and Bartlett’s sphericity test (*X*2 = 4,130, *p* < 0.001) allow for the implementation of a principal component analysis (PCA). Seven factors were highlighted based on the configuration of a component scree plot (Figure 1).

Loadings higher than 0.4 were accepted for analysis. Since factor 7 contained only two items in the structure, the team decided to exclude this factor from further analysis. At this stage, the questionnaire contained 41 items.

#### 3.1.3. The discriminatory power of the items (Cronbach’s alpha coefficient)

As a result of the analysis, two items were removed from the ‘multilingual and multicultural skills’ section. However, no further items were removed because all the test items correlated to the overall result on a level higher than 0.2. Ultimately, the structure of the diagnostic tool (39 items) appeared in the following form (Table 1): CS: Communication skills (12 items: *α* = 0.823); MMS: Multilingual and multicultural skills (5 items: *α* = 0.799); DS: Digital skills (4 items: *α* = 0.780); ES: Entrepreneurial skills (7 items: *α* = 0.659); CCAE: Competences in cultural awareness and expression (7 items: *α* = 0.594); and SCS: Social and Civic skills (4 items: *α* = 0.616).

**Table 1:** Accuracy coefficient of the AENI questionnaire measurements (*N* = 210).

	Number of items	*M*	*SD*	Cronbach’s alpha
CS	12	3.83	0.60	0.823
MMS	5	3.46	0.98	0.799
DS	4	3.94	0.79	0.780
ES	7	3.40	0.66	0.659
CCAE	7	3.85	0.57	0.594
SCS	4	3.74	0.70	0.616
AENI	39	3.65	0.36	0.846

AENI, adult education needs inventory; CS, communication skills; MMS, multilingual and multicultural skills; DS, digital skills; ES, entrepreneurial skills; CCAE, competences in cultural awareness and expression; SCS, social and civic skills; *M*, mean; *SD*, standard deviation.

Cronbach’s alpha coefficient.

Each of the singled out-areas proved to had a satisfactory level of diagnostic accuracy. What is significant is that the complete tool also features a high coefficient of accuracy. In this case, Cronbach’s alpha was 0.803.

### 3.2. Internal and theoretical accuracy

#### 3.2.1. Description of the research sample

The sample consisted of 98 random participants (16 male and 83 female). Participants were selected for the second measurement based on their professional (…). Similar to the first measurement, the second test was conducted in an auditory survey of small groups of workers. The authors informed participants about the aim and potential outcome of the study and signaled refusal of participation in the study by handing back empty test questionnaires. During the survey, 98 questionnaires were distributed, and 98 were collected back; thus, the response rate was 100%. They were engaged in professional, semi-professional, or amateur activities in the third sector. Previously calculated minimal sample size of 138 observations, when compared with the collected 98 observations, gave a possibility to calculate maximum bias, which was not higher than 5%; thus, a 95% confidence interval was possible to sustain.

#### 3.2.2. Internal accuracy

The Confirmatory Factor Analysis (CFA) has been conducted to check internal consistency. The created model proved significant (*X*2 = 1,200, *p* < 0.001). Fit measures were inconclusive (CFI = 0.580, TLI = 0.547, SRMR = 0.108, and RMSEA = 0.087) and suggested that the model had significant measuring value, although there is room for improvement in the future (Xia and Yang, 2019). Factor loadings (Table 2) proved in the majority of indicators significant. Internal factor covariances were noted (Table 3), although those were assumed to occur due to the close relationship between individual factors. An inter-correlation between sets of variables for the AENI questionnaire was performed to check its internal accuracy (Table 4).

**Table 2:** Confirmatory factor analysis factor loadings summary.

				95% Confidence interval			
Factor	Indicator	Estimate	SE	Lower	Upper	*Z*	*p*
CS	1	0.6487	0.1083	0.43641	0.861	5.9894	< 0.001
	2	0.3136	0.0916	0.13408	0.493	3.4236	< 0.001
	4R	0.6361	0.1144	0.41191	0.860	5.5614	< 0.001
	5	0.3223	0.0921	0.14174	0.503	3.4985	< 0.001
	6	0.6183	0.0966	0.42892	0.808	6.3993	< 0.001
	7	0.7438	0.0867	0.57395	0.914	8.5836	< 0.001
	8	0.4825	0.1070	0.27283	0.692	4.5104	< 0.001
	24	0.3679	0.0897	0.19214	0.544	4.1030	< 0.001
	25R	0.6407	0.1070	0.43104	0.850	5.9901	< 0.001
	30	0.5204	0.0684	0.38623	0.654	7.6040	< 0.001
	31	0.5029	0.1018	0.30335	0.702	4.9395	< 0.001
	36R	0.1621	0.1086	−0.05071	0.375	1.4929	0.135
MMS	9	0.7551	0.1368	0.48700	1.023	5.5203	< 0.001
	11	0.8830	0.1122	0.66312	1.103	7.8715	< 0.001
	12	0.9343	0.1113	0.71611	1.153	8.3914	< 0.001
	13	0.7329	0.1137	0.51009	0.956	6.4477	< 0.001
	37	0.2231	0.1153	−0.00279	0.449	1.9357	0.053
DS	20	0.5743	0.0668	0.44347	0.705	8.6020	< 0.001
	21	0.7638	0.0784	0.61010	0.918	9.7390	< 0.001
	22	0.7032	0.0940	0.51889	0.887	7.4787	< 0.001
	23	0.3415	0.0493	0.24500	0.438	6.9341	< 0.001
ES	16	0.4068	0.0904	0.22963	0.584	4.5005	< 0.001
	17R	0.3691	0.1234	0.12720	0.611	2.9905	0.003
	19	0.8997	0.1172	0.66997	1.129	7.6763	< 0.001
	26R	0.1488	0.1289	−0.10375	0.401	1.1549	0.248
	33R	0.8050	0.1189	0.57192	1.038	6.7686	< 0.001
	34R	0.3963	0.1029	0.19472	0.598	3.8530	< 0.001
	35R	0.6202	0.1275	0.37034	0.870	4.8653	< 0.001
CCAE	10R	0.1790	0.1171	−0.05043	0.409	1.5292	0.126
	14R	0.0781	0.1851	−0.28463	0.441	0.4222	0.673
	15R	0.0142	0.1933	−0.36457	0.393	0.0736	0.941
	27R	0.3899	0.1715	0.05372	0.726	2.2732	0.023
	32R	0.4978	0.1758	0.15324	0.842	2.8315	0.005
	38R	0.3334	0.1523	0.03486	0.632	2.1888	0.029
	39R	0.5297	0.1680	0.20042	0.859	3.1528	0.002
SCS	3	0.2788	0.1022	0.07849	0.479	2.7278	0.006
	18	0.4223	0.1024	0.22164	0.623	4.1249	< 0.001
	28	0.6955	0.1444	0.41243	0.979	4.8157	< 0.001
	29	0.6985	0.1341	0.43573	0.961	5.2097	< 0.001

AENI, adult education needs inventory; CS, communication skills; MMS, multilingual and multicultural skills; DS, digital skills; ES, entrepreneurial skills; CCAE, competences in cultural awareness and expression; SCS, social and civic skills; *M*, mean; *SD*, standard deviation.

R-reversed scale.

**Table 3:** Confirmatory factor analysis factor covariances summary.

				95% Confidence interval			
		Estimate	SE	Lower	Upper	*Z*	*p*
CS	MMS	0.259	0.1164	0.0312	0.487	2.22758	0.026
	DS	0.179	0.1163	−0.0488	0.407	1.54053	0.123
	ES	0.191	0.1254	−0.0550	0.436	1.52115	0.128
	CCAE	0.279	0.1565	−0.0278	0.586	1.78261	0.075
	SCS	0.759	0.0961	0.5702	0.947	7.89493	< 0.001
MMS	DS	0.162	0.1197	−0.0723	0.397	1.35604	0.175
	ES	0.000	0.1301	−0.2548	0.255	0.00198	0.998
	CCAE	0.238	0.1682	−0.0920	0.567	1.41294	0.158
	SCS	0.326	0.1356	0.0603	0.592	2.40493	0.016
DC	ES	0.131	0.1239	−0.1123	0.373	1.05358	0.292
	CCAE	0.337	0.1421	0.0584	0.615	2.37084	0.018
	SCS	0.159	0.1566	−0.1482	0.466	1.01379	0.311
EC	CCAE	0.124	0.1700	−0.2092	0.457	0.72947	0.466
	SCS	0.350	0.1393	0.0766	0.623	2.50982	0.012
CCAE	SCS	0.220	0.2168	−0.2044	0.645	1.01689	0.309

AENI, adult education needs inventory; CS, communication skills; MMS, multilingual and multicultural skills; DS, digital skills; ES, entrepreneurial skills; CCAE, competences in cultural awareness and expression; SCS, social and civic skills; *M*, mean; *SD*, standard deviation.

**Table 4:** Inter-correlations for AENI (*N* = 98) measurement and overall result.

		1.	2.	3.	4.	5.	6.	7.
1.	CS	–						
2.	MMS	0.267^**^	–					
3.	DS	0.168	0.194	–				
4.	ES	0.264^**^	0.070	0.061	–			
5.	CCAE	0.149	0.087	0.076	−0.021	–		
6.	SCS	0.527^***^	0.299^**^	0.154	0.220^*^	0.114	–	
7.	AENI	0.902^***^	0.427^***^	0.286^**^	0.463^***^	0.325^**^	0.606^***^	–

AENI, adult education needs inventory; CS, communication skills; MMS, multilingual and multicultural skills; DS, digital skills; ES, entrepreneurial skills; CCAE, competences in cultural awareness and expression; SCS, social and civic skills; *M*, mean; *SD*, standard deviation; *p*, level of statistical significance.

^*^p < 0.05, ^**^p < 0.01, ^***^p < 0.001.

The highest correlations occur between individual measurements and the overall AENI result. Most measurements do not correlate with one another, except the CS and SCE measurements. However, in this case, it is justifiable because social and civic skills require communication skills, even though they are not the same. To sum up, analysis of the inter-correlation proves an acceptable homogeneity of the measurements.

#### 3.2.3. Theoretical accuracy

Each of the research subjects completed two questionnaires: Adult Education Needs Inventory (AENI) and Social Skills Profile (PROKOS) [original polish title of PROKOS test: Profil Kompetencji Społecznych]. PROKOS is a test which measures the level of such social skills as assertiveness, cooperativeness, sociability, social resourcefulness, and social activeness. This tool is used to assess and recruit staff (Martowska and Matczak, 2013).

Three hypotheses were formulated, which concerned the overall results (AENI and PROKOS questionnaires) and the selected ranges to check the theoretic accuracy.

*Hypothesis 1*: The overall score for educational needs in the AENI questionnaire correlates to the overall score for social skills in the PROKOS questionnaire.

*Hypothesis 2*: Both the overall score and individual skills in the PROKOS questionnaire correlate to the measurement of ‘communication skills’ in the AENI questionnaire.

*Hypothesis 3*: Both the overall score and individual skills in the PROKOS questionnaire correlate to the measurement of ‘social and civil skills’.

Burns (1985) and Paterson (2000) have, among others, pointed to the connection between social and communication skills. Generally, communication skills are treated as a part of social skills (Kobylarek, 2009) or as a basis for formulating other interpersonal skills (Warzocha, 2016).

#### 3.2.4. Descriptive statistics

Before calculating the correlations, the normality of the distribution of both tests was checked. In the AENI questionnaire, MMS, DS, and SCS did not meet the requirements of a normal distribution, and in the PROKOS questionnaire, the scores for co-operative skills did not meet the requirements of a normal distribution (Tables 5 and 6).

**Table 5:** Normality of distribution of the AENI (*N* = 98).

	CS	MMS	DS	ES	CCAE	SCS	AENI
*M*	46.9	18.4	16.7	24.3	27.2	15.4	29.3
*SD*	6.66	4.08	2.61	4.44	3.46	2.56	2.95
*p*	0.380	0.001	<0.001	0.205	0.133	0.005	0.847

AENI, adult education needs inventory; CS, communication skills; MMS, multilingual and multicultural skills; DS, digital skills; ES, entrepreneurial skills; CCAE, competences in cultural awareness and expression; SCS, social and civic skills; *M*, mean; *SD*, standard deviation; *p*, level of statistical significance.

Shapiro–Wilk normality scores.

**Table 6:** Normality of distribution of the PROKOS (*N* = 98).

	Assertiveness skills	Co-operative skills	Friendship skills	Social resourcefulness	Social skills	PROKOS
*M*	40.31	54.5	33.7	41.8	17.2	187
*SD*	6.59	5.77	5.79	4.82	3.08	22.2
*p*	0.089	0.002	0.105	0.105	0.113	0.318

PROKOS, social skills profile (pol. Profil Kompetencji Społecznych); *M*, mean; *SD*, standard deviation; *p*, level of statistical significance.

Shapiro–Wilk normality scores.

In order to provide a more detailed description of the scales, descriptive statistics are also presented in Table 5. Each research subject gave their replies on a scale from 1 to 5. The higher the mark, the higher the measurement in each scale. It was possible to gain a maximum of 195 points in the questionnaire and a minimum of 39. The most points could be achieved in the area of Communication Skills, which forms one of the most important competences necessary in adult education. A proposal for calculating the scores is presented in Table 7.

**Table 7:** Descriptive statistics of AENI and PROKOS (*N* = 98).

	Min	Max	*M*	*SD*	Slant	Kurtosis
CS	12	60	46.9	6.66	−0.04	−0.26
MMS	5	25	18.4	4.08	−0.24	−0.93
DS	4	20	16.7	2.61	−0.67	0.52
ES	7	35	24.3	4.44	−0.27	0.28
CCAE	7	35	27.2	3.46	−0.08	−0.28
SCS	4	20	15.4	2.56	−0.05	−0.08
AENI	39	195	149	13.9	0.102	0.313

AENI, adult education needs inventory; PROKOS, social skills profile (pol. Profil Kompetencji Społecznych); CS, communication skills; MMS, multilingual and multicultural skills; DS, digital skills; ES, entrepreneurial skills; CCAE, competences in cultural awareness and expression; SCS, social and civic skills; Min, minimum value, Max, maximum value; *M*, mean; *SD*, standard deviation.

Correlation between AENI and PROKOS scores.

In view of the fact that the normality of distribution of three scales in AENI, as well as three scales in the PROKOS questionnaire, did not meet normality requirements (Table 8), the correlation between variables measured with these tools was tested with the nonparametric Spearman’s correlation test.

**Table 8:** Coefficient values in the correlation of measurements between AENI and PROKOS (*N* = 98).

	PROKOS–social skills					
	Assertiveness skills	Co-operative skills	Friendship skills	Social resourcefulness	Social skills	PROKOS
CS	0.507^***^	0.665^***^	0.673^***^	0.533^***^	0.593^***^	0.693^***^
MMS	0.234^*^	0.236^*^	0.158	0.203^*^	0.154	0.195
DS	0.282^**^	0.257^*^	0.147	0.189	0.240^*^	0.226^*^
ES	0.292^*^	0.341^*^	0.231^*^	0.132	0.355^***^	0.306^*^
CCAE	0.020	0.167	0.152	0.095	0.058	0.128
SCS	0.504^***^	0.385^***^	0.458^***^	0.391^***^	0.543^***^	0.501^***^
AENI	0.553^***^	0.702^***^	0.646^***^	0.527^***^	0.636^***^	0.703^***^

AENI, adult education needs inventory; PROKOS, social skills profile (pol. Profil Kompetencji Społecznych); CS, communication skills; MMS, multilingual and multicultural skills; DS, digital skills; ES, entrepreneurial skills; CCAE, competences in cultural awareness and expression; SCS, social and civic skills.

^*^p < 0.05, ^**^p < 0.01, ^***^p < 0.001.

The results confirm the hypotheses which predict a correlation between the overall scores in both questionnaires and the measurements relating to communication and social skills. Furthermore, the ‘communication skills’ in the AENI questionnaire correlate with all the social skills measured in the PROKOS questionnaire, and the same is true for the measurement of “Social and Civic Skills.” Therefore, the AENI questionnaire has a satisfactory level of theoretical accuracy.

### 3.3. Application

The adult education sector needs more research tools, which is a significant barrier to developing andragogic research. At the same time, this situation is blocking the development of andragogic theory and, what goes with it, andragogy itself. If such tools are to be applied universally, they should be prepared by professionals and properly tested, and then published as open-source so that they could be used by adult education instructors, who are rarely authorized to use professional psychological tools. The tool proposed is an attempt to overcome such developmental barriers in andragogy. The development of this research tool enables a quick and relatively easy diagnosis of a basic set of key competences. It can also be used by every adult education instructor because it is universally available, together with an overview and set of instructions.

The AENI tool can be used by adult educators (self-testing and calculating the results) to identify areas worth developing. The tool can also be used by employers to diagnose the needs of their educators, in order to plan training to improve competences with lower scores.

The AENI questionnaire is a tool for researching key competences, which furthers the concept proposed by the Council of the European Union in 2018. It possesses satisfactory psychometric properties—high reliability and proven accuracy. The universality of the tool comes from the fact that it is intended to be used in researching educational needs by everybody working in the adult education sector in its broadest sense—both in private firms and in public institutions.

As a result of the analysis, 6 areas of need were highlighted. Low scores on the scale mean that a respondent needs training in that area because that skill is not well developed. Such information is vital for both the employer and educator who wants to discover the areas which need improvement.

### 3.4. Key competence areas

As a result of the analysis, the following key competence areas were defined:

#### 3.4.1. Communication skills (CS)

Those who score high on this scale like to engage in a discussion and can carry on a conversation in any situation. They feel at ease during a conversation, send out clear and understandable communications, and have no problem understanding their interlocutor or expressing thoughts, which they are able to communicate unconventionally. Such people are able to work as part of a team, motivate others, share information, and be successful negotiators.

#### 3.4.2. Multilingual and multicultural skills (MMS)

Those who score high on this scale know at least one foreign language, use it every day, and make use of several opportunities to learn a new language. They also manifest an interest in various cultures.

#### 3.4.3. Digital skills (DS)

Those who score high on this scale willingly take advantage of technological innovations and make use of them to communicate effectively. They feel proficient with ICT and have no problem making use of various digital tools and software.

#### 3.4.4. Entrepreneurial skills (ES)

Those who score high on this scale recognize the necessity for good planning and try to proceed according to a plan. They feel competent and efficient when carrying out their tasks. They are able to form an orderly relationship with the team, as well as implement and monitor the tasks delegated to them.

#### 3.4.5. Competences in cultural awareness and expression (CCAE)

Those who score high in CCAE are open to continuous learning, including improving their foreign language skills. They also appreciate the European Union’s integration policies, such as recognizing cultural differences. They perceive the value of contemporary culture and can use the arts to express themselves.

They do not trust “conspiracy theories” but use science to reach the truth. They also see many possibilities conducive to realizing their ideas.

#### 3.4.6. Social and civic skills SCS)

Those who score high on this scale use different sources when planning and looking for information. This is because they feel they have some influence over what is going on around them and engage in social affairs which are important to them.

## 4. Discussion

The AENI questionnaire presented in the article examines six competences that can create an attitude to educate an adult in a changing reality effectively. It is a tool that can be used in adult education to identify areas of improvement for educators. The proposed tool fits into the concept of lifelong education, which contemporary international organizations define very broadly as the field of policy, practice, research, and science, and how it is shaped by different philosophies of education and understandings of human nature and ethical principles (UNESCO, 2021). The American Association for Adult and Continuing Education (AAACE) emphasizes that lifelong learning contributes to human self-actualization and positive social change (Hunter-Johnson et al., 2021).

There are many challenges facing modern adult educators. In a world of changing knowledge, the person who teaches should be able to acquire this knowledge and make it interesting. In the literature, there are many classifications of key educators’ competences. One of them, for example, presents such competences as IT competence, creative/cognitive competence, intercultural competence, ability to work in virtual, multicultural teams, emotional and social intelligence, and interdisciplinary competence (Przytuła, 2018). What most classifications have in common are the needs arising firstly from the development and increasing use of new technologies and secondly from globalization, migration, and, therefore, the ability to find oneself in multiculturalism. Trainers working with refugees sometimes do not feel sufficiently prepared for such work because they somehow did not imagine this is precisely the kind of educational demand they will have to respond to. Research on refugee education shows that most trainers want to educate themselves on basic principles of adult and vulnerable group education, diversity and interculturalism issues, and psychology (Kafritsa et al., 2021).

Another challenge for which adult educators were unprepared and demonstrated the importance of adapting to new situations is the work of educators during a pandemic. The pandemic caused many areas of education to go online. The authors note that “digital adult education” became one of the key competences of both educators and their students (Petrushenko et al., 2021). Nevertheless, it was more than just technical skills that were important here. In an article, researchers on this issue found that educators have developed a range of innovative and dynamic social solidarity-oriented pedagogies that can contribute to more equitable and inclusive socio-technological relationships in a post-pandemic future (Smythe et al., 2021). Motivating, interest, and sometimes social support for adults in general and during a pandemic is a major challenge. Research shows that adult educators need up-to-date, innovative, interesting, and engaging materials to help develop adult learners’ creativity (Boghian et al., 2022). So, what resounds in both the context of interculturalism and new technologies is flexibility, innovation, and the ability to make adult learners curious.

It should be emphasized that the key competences of an adult are characterized by the fact that they refer to universal skills and knowledge (Põlda et al., 2021). Having them becomes the foundation for getting actual knowledge on an ongoing basis and dealing with the adult-educator relationship. Morelli (2022) points out that the relevant competences are the capacity to innovate, create, problem-solve and collaborate through horizontal leadership with teams and individuals across the organization.

In the digital society, the possession of knowledge is no longer enough as access to it becomes more and more egalitarian. Researchers point to a change in the professional competences of adults, which are characterized by a shift from the paradigm of knowledge to competences related to motivation and the ability to assess self-esteem (Sin et al., 2020).

Researchers consistently emphasize the need for lifelong learning, which results, inter alia, from the globalization of the market and the development of new technologies (Yamashita et al., 2019). It is predicted that some occupations will cease to be needed, whereas others will require the employee’s ability to adapt to changes and skills related to fitting into international teams and efficient use of new technologies (Przytuła, 2018).

The concept of lifelong learning shines through many European education and research programs (Bohnenberger, 2022). It should be emphasized, however, that the vast majority are directed at people from the risk group, who are subject to an exclusion (Pinto et al., 2016).

Meanwhile, the desire to satisfy specific learning needs is also reported by adult learners (Owusu-Agyeman et al., 2018) and by educators wanting to develop their academic skills and use the best educational practices (Czerniawski et al., 2017).

Research is ongoing in measuring educational needs and their identification and specification (Oberländer et al., 2020). This was also the aim of the authors of the AENI tool.

Considering the skills that emerged and the educational needs that are integral to them, the insofar research can be divided into 3 groups.

Researchers usually explore educational needs regarding the areas of skill that have little in common with the systematics being proposed. Some of the most important examples of such explorations are:

- classifications referring to old education reports and predictions regarding the course of social development. Radovan (2019) refers above all to the skills and competences of the future, further defined as “learning to learn” in a report by Faure (1979);
- specific skills useful in particular sectors, such as planning, and the skills needed to formulate a balanced development (Hakio and Mattelmäki, 2019).
- extra-European classifications of skills that relate to somewhat different educational realities, determined by diverse political, organizational, and cultural influences, such as creative thinking (Hipkins, 2018).

Much research, which does not consider the Council of the European Union model, distinguishes the same or similar areas, often defined in different ways. An example is a work of Głomb (2020), which emphasizes the role of digital skills but is defined in such a way that they can be identified partly with the areas of digital skills highlighted by the AENI questionnaire and partly with the area of communication skills.

Only a small portion of research refers directly to the same model, which constituted the starting point for the skill sets proposed here for researching educational needs (Petrėtienė et al., 2020). Deficiencies in this area should not be surprising because the model has only been functioning for a short time. Therefore, scientific discussion over its accuracy and substantiation may last some time. Work on the proposed tool revealed, for example, that some of the skill areas identified by the Council of the European Union should be redefined during the research because they have been aggregated differently.

We are aware that the competences indicated in the questionnaire: *Competences in cultural awareness and expression; Entrepreneurial skills; Digital skills; Multilingual and multicultural skills; Communication skills–may not cover all the necessary areas for the development of adult educators*. However, analyzing the literature and based on the statistical analysis with which we examined the content analysis areas selected from the theory, we believe that the competences identified in the questionnaire can be described as key. They take into account two important trends of catalysts of change in contemporary reality: the development of new technologies and interculturalism. Therefore, such competences, which are universal and at the same time take into account the dynamics of modernity, can be used to shape further competences and the acquisition of skills and knowledge corresponding to current educational needs.

## 5. Conclusion

Presented in this study AENI questionnaire supports the idea of key competences created by the Council of the European Union in 2018. AENI has good psychometric properties verified by high reliability and verified fitness. Low scores indicate that respondents are in high need of education due to low competences in a given area. Therefore, test results can be important for the employee and the educator because they indicate which areas require development.

Recent events have shown that we sometimes need more time to prepare for changes regarding both the form of education and the educational needs of adults. For example, the rapid growth and spread of online education forced by a pandemic, or the educational needs of refugees trying to find their way in a culturally different reality, are huge challenges for those working with adults. To deal with such challenges, the nature of the owned competence should be universal.

## Data availability statement

The raw data supporting the conclusions of this article will be made available by the authors, without undue reservation.

## Ethics statement

Ethical review and approval was not required for the study on human participants in accordance with the local legislation and institutional requirements. The patients/participants provided their written informed consent to participate in this study.

## Author contributions

LŚ: conceptualization, methodology, software, validation, formal analysis, resources, data curation, writing—original draft, writing—review and editing, supervision, project administration, and funding acquisition. KB and AK: conceptualization, methodology, software, validation, formal analysis, resources, data curation, writing—original draft, and writing—review and editing. MŠ: conceptualization, validation, resources, writing—review and editing, and visualization. All authors of this manuscript meet the authorship criteria according to the latest guidelines of the International Committee of Medical Journal Editors (ICMJE). All authors contributed to the article and approved the submitted version.

## Funding

The research was organized and conducted by Foundation Pro Scientia Publica from the finances of the European Union within Erasmus+ project titled *Needs of adults education stakeholders*, no.: 2019-1-PL01-KA204-065792).

## Conflict of interest

The authors declare that the research was conducted in the absence of any commercial or financial relationships that could be construed as a potential conflict of interest.

## Publisher’s note

All claims expressed in this article are solely those of the authors and do not necessarily represent those of their affiliated organizations, or those of the publisher, the editors and the reviewers. Any product that may be evaluated in this article, or claim that may be made by its manufacturer, is not guaranteed or endorsed by the publisher.
