# Supplementary material for: Adult education needs inventory: Construction and application
Source: Front Psychol. 2022 Dec 23;13:1035283. doi: 10.3389/fpsyg.2022.1035283 (PMC9816405; doi:10.3389/fpsyg.2022.1035283)
Supplement: Supplementary file 1 [file Table_1.DOCX]

**Attachment-questionnaire (key)**

*2021/01/07*

*Authors: Luba Jakubowska, Aleksander Kobylarek, Kamil Błaszczyński*

ERASMUS+ PROGRAMME

KEY ACTION 2: COOPERATION FOR INNOVATION

Strategic Partnership in the field of Adult Education

Project: Needs of adults’ education stakeholders

N° 2019-1-PL01-KA204-065792

**AENI**

(Adults Education Needs Inventory)

*Questionnaire to study training needs in the field of key competences of adults*

Below is a list of 39 statements. Please indicate how far you agree with each statement on a scale of 1 to 5 (**1 = decidedly do not agree, 5 = decidedly agree**). There are no good or bad or right or wrong answers. Do not spend too much time on each statement, but give the answer which first comes to mind. This research is completely anonymous, and the results will be used exclusively for scientific purposes.

**NOTE**: Please give answers to all the statements

| **1 = decidedly do not agree, 5 = decidedly agree** | | **1** | **2** | **3** | **4** | **5** |
| --- | --- | --- | --- | --- | --- | --- |
| 1 | **CC** I can have a conversation in any situation (e.g., in a conflict, or when my interlocutor has a different opinion) |  |  |  |  |  |
| 2 | **CC** I consider that I can send clear and understandable messages |  |  |  |  |  |
| 3 | **SCC** When seeking information, I try to use various sources |  |  |  |  |  |
| 4 R | **CC** Very often I experience a situation when it is difficult to express my thoughts |  |  |  |  |  |
| 5 | **CC** I usually don’t have a problem in understanding my interlocutor |  |  |  |  |  |
| 6 | **CC** I enjoy discussing with various people |  |  |  |  |  |
| 7 | **CC** I feel at ease when conversing with other people |  |  |  |  |  |
| 8 | **CC** I can express my thoughts in an unconventional way |  |  |  |  |  |
| 9 | **MM**I am fluent in speaking and writing more than one foreign language |  |  |  |  |  |
| 10R | **OSC** I consider that I do not need to develop my knowledge of foreign languages |  |  |  |  |  |
| 11 | **MM** I seize different opportunities in learning foreign languages |  |  |  |  |  |
| 12 | **MM** I like learning new languages |  |  |  |  |  |
| 13 | **MM** I use a foreign language every day (e,g,, watching films, reading books) |  |  |  |  |  |
| 14R | **OSC** I consider that maths do not help in uncovering the truth |  |  |  |  |  |
| 15R | **OSC** There are justifiable doubts concerning certain theories, e.g., human influences the climate, or the use of vaccination |  |  |  |  |  |
| 16 | **EC** I consider that effective action requires a clear and unambiguous plan |  |  |  |  |  |
| 17R | **EC** I sometimes act illogically |  |  |  |  |  |
| 18 | **SCC** I often use various sources when planning |  |  |  |  |  |
| 19 | **EC** I generally follow a pre-determined plan |  |  |  |  |  |
| 20 | **DC** I know how to use the new technologies for more effective communication |  |  |  |  |  |
| 21 | **DC** I use various technological innovations |  |  |  |  |  |
| 22 | **DC** I feel that I am competent enough in information technology |  |  |  |  |  |
| 23 | **DC** I can easily use the most common devices (PC, smartphone, laptop, tablet, etc.) |  |  |  |  |  |
| 24 | **CC** I can work in a group |  |  |  |  |  |
| 25R | **CC**I think that it is difficult for me to form relationships with other people |  |  |  |  |  |
| 26R | **EC** I am sometimes difficult in relationships with other people |  |  |  |  |  |
| 27R | **OSC** It is impossible to reconcile EU integration policy with cultural distinctiveness |  |  |  |  |  |
| 28 | **SCC** I engage in social issues which are important to me |  |  |  |  |  |
| 29 | **SCC** I feel that I have an effect on my surroundings |  |  |  |  |  |
| 30 | **CC** I can motivate others to realize common aims |  |  |  |  |  |
| 31 | **CC**I am effective in negotiations |  |  |  |  |  |
| 32R | **OSC** The world does not offer many possibilities to realise my ideas |  |  |  |  |  |
| 33R | **EC** I don’t like the planning stage |  |  |  |  |  |
| 34R | **EC** I consider that controlling and monitoring work are generally pointless |  |  |  |  |  |
| 35R | **EC** I sometimes have a feeling of my own mismanagement when carrying out a task |  |  |  |  |  |
| 36R | **CC** I rarely give feedback to my co-workers |  |  |  |  |  |
| 37 | **MM** I am interested in various cultures |  |  |  |  |  |
| 38R | **OSC** I would not be able to express myself in any form of art |  |  |  |  |  |
| 39R | **OSC** For me, contemporary culture is worthless |  |  |  |  |  |

**CC** Communication competences (max. 60 pts.): 1, 2, 4R, 5, 6, 7, 8, 24, 25R, 30, 31, 36R

**MM** Multilingualism and multiculturalism (max. 25 pts.): 9,11,12, 13, 37,

**DC** Digital competences (max. 20 pts.): 20, 21, 22, 23

**EC** Entrepreneurship competences (max. 35 pts.): 16, 17R, 19, 26R, 33R, 34R, 35R

**OSC** Openness to science and culture (max. 35 pts.): 10R, 14R, 15R, 27R, 32R, 38R, 39R

**SCC** Social and civil competences (max. 20 pts.): 3, 18, 28, 29

**R** Reverse score

**Interpretation of the results**

Standardization research is currently being carried out on a group representing educators from various European countries. At this stage the authors propose the use of interim norms to help in converting the raw data. Depending on the number of points gained in each particular class, it is possible to allocate the results to one of three groups – high, medium and low. Low results indicate a low level of competence in a particular educator, and therefore an increased need to improve that competence.

|  | **low** | **medium** | **high** |
| --- | --- | --- | --- |
| **CC** | 12 -31 | 32-41 | 42-60 |
| **MM** | 5-13 | 14-17 | 18-25 |
| **DC** | 4-10 | 11-14 | 15-20 |
| **EC** | 7-18 | 19-24 | 25-35 |
| **OSC** | 7-18 | 19-24 | 25-35 |
| **SCC** | 4-10 | 11-14 | 15-20 |
